# LaksData: Comprehensive database of salmon fillets images for use in vision-based food automation applications

**DOI:** 10.1016/j.dib.2026.112771

**Published:** 2026-04-11

**Authors:** Abhaya Pal Singh, Michael Angelo Amith Fenelon, Jens Petter Wold, Antonio Candea Leite

**Affiliations:** a Realtek, Department of Robotics, Norwegian University of Life Sciences, (NMBU), P.O. Box 5003, Ås 1432, Norway; b Norwegian Institute of Food, Fisheries and Aquaculture Research, P.O. Box 6122, Tromsø 9291, Norway

**Keywords:** Salmon fillets, Robotics, Computer vision, Image processing, Data annotations

## Abstract

This article provides a salmon fillet dataset to investigate the detection of distinct regions, undesirable spots, and possibly the higher nutrient content measurements. Since we know that the belly of salmon is high in omega-3 fatty acids, we can use computer vision and image processing to identify the belly areas of salmon fillets (for trim A, B, and C cuts, trim A cut has the largest belly area) and determine the percentage of these fatty acids. As a result, this dataset becomes essential for training models that identify and examine the belly regions. Datasets were acquired from Lerøy Aurora, a salmon processing plant in Skjervøy, Norway, as well as images taken in our lab during experiments. To acquire the images at the Lerøy plant, two settings were used: (i) using a stand with 3 Intel RealSense RGB-D cameras and (ii) using a stand with 1 Intel RealSense RGB-D camera, depending on the amount of space available to put our setup near the production line. The camera equipment was positioned close to the production line. In total, 712 RGB images, 10 ROS (Robot Operating System) bags with 3 camera settings, and 5 ROS bags with 1 camera setting were taken in the Lerøy plant, while 60 RGB images were captured at the NMBU lab. ROS nodes were utilized to capture both the ROS bags (which carried RGB-D information) and the RGB images. To facilitate further research on salmon fillets, this collection also contains 509 multispectral images of fish fillets. The dataset is intended primarily as a benchmarking and pre-training resource, demonstrating the potential of computer vision for salmon fillet analysis. In conclusion, this comprehensive dataset provides a solid base for potential research on automated salmon fillet analysis. This will enable computer vision and image processing to enhance quality control and nutritional evaluation of salmon fillets.

Specifications Table

**Table:** 

Subject	Computer Sciences
Specific subject area	Computer vision for food automation.
Type of data	Raw, ROS bags with RGB and depth data, RGB Images, Multipectral Images, JSON files
Data collection	The ROS bags are captured in two different settings: (i) Three Intel RealSense depth cameras, viz., two D415 for left and right view and one D435 for top view of the trimmed fillets; (ii) One Intel RealSense depth camera for the top view of the untrimmed fillet. The RGB images have also been captured for the top view of the fillets using one D435 camera. The camera equipment was located near to the production line, with the cameras 0.7 m above the conveyor. In total, 712 RGB images, 10 ROS (Robot Operating System) bags with 3 camera settings, and 5 ROS bags with 1 camera setting were taken in the Lerøy plant, while 60 RGB images were captured at the NMBU lab.
Data source location	The data is collected at Lerøy Aurora plant, Strandveien 4, 9180, Skjervøy, Norway: 70.0360887966681, 20.99002555556582 and NMBU Lab, Ås, Norway: 59.66553843295463, 10.777997474303957
Data accessibility	**Repository name:** Dataverse.no **Direct URL to data:**https://dataverse.no/privateurl.xhtml?token=9d73e45d–2458–4c37–97f2–2059ce00f403
Related research article	None

## 1 Value of the Data

• **Quality Assessment:** This dataset can support the development of automated fillet quality assessment systems in the fish industry. Using accurate annotations and a diverse image collection of salmon fillets, deep-learning models can be trained or fine-tuned to detect specific quality-related regions such as the belly area or unwanted spots (blood and melanin). Since the images were captured in a real industrial environment using both single- and multi-camera configurations, the dataset reflects authentic variations in lighting and surface conditions, providing a realistic basis for model evaluation and benchmarking. These models can later be integrated into sorting and grading systems in processing facilities to categorize fillets and enhance packing efficiency.
• **Monitoring and Control:** The dataset enables image-processing applications that can detect and classify unwanted spots in salmon fillets, supporting quality control and consistency in industrial production. Because the data were collected under real factory lighting and handling conditions, it provides a valuable foundation for testing how computer vision methods perform under natural variations. This can also support the development of monitoring systems that ensure product uniformity and assist in traceability and health compliance.
• **Research and Development (R&D):** Research groups and industry can use this dataset to explore and develop robotics, image processing, and computer vision techniques focused on salmon fillets. The inclusion of belly region, blood, and melanin spot annotations allows researchers to experiment with segmentation, classification, and detection models. Although only a limited number of annotated images (32) are provided, the dataset serves as a valuable benchmarking and pre-training resource that can be expanded with additional data or used for transfer learning in related studies.
• **Education:** The dataset can be used by educators and instructors to design teaching tools and hands-on exercises in image processing, machine learning, and food technology. It provides students with access to real industrial data, allowing them to practice segmentation, classification, and computer vision methods on authentic salmon fillet images. The availability of both RGB data from multiple camera setups and expert annotations enhances its value as a teaching and demonstration resource.

## 2 Background

Salmon farming, a vital part of the worldwide fish industry, is led by Norway. As of 2022, the total value of Norwegian Atlantic salmon exports was $11.2 billion. Norwegian salmon is the world’s most popular and sought-after fish, according to a Norsk Sjømatråd survey [1] and in 2023, Norway exported fish worth NOK 171.7 billion (about $16 billion) [2]. Production also presents some challenges that must be overcome to further improve the efficiency of the fish industry. In the salmon industry, unwanted spots such as blood (red) and melanin (black) can impact consumer perception of quality and thus lower the selling price [3]. In 2003, 7% of salmon were affected, and by 2015, it had increased to 20% [4]. Paper [6,7] demonstrated robotic and computer vision systems for determining omega-3 fatty acid levels and detecting salmon belly regions. The enhanced setup efficiently planned robot mobility by utilizing a hand-to-eye camera arrangement and open-source AI. Other studies [8,9] demonstrated computer vision's ability to detect melanin spots and classify salmon by size, shape, and colour, increasing efficiency and lowering manual labour in quality evaluation.

Therefore, this dataset is essential for identifying potential issues and enhancing the quality evaluations of fish fillets for both researchers and the fish industry.

## 3 Data Description

**Fig. 1:**
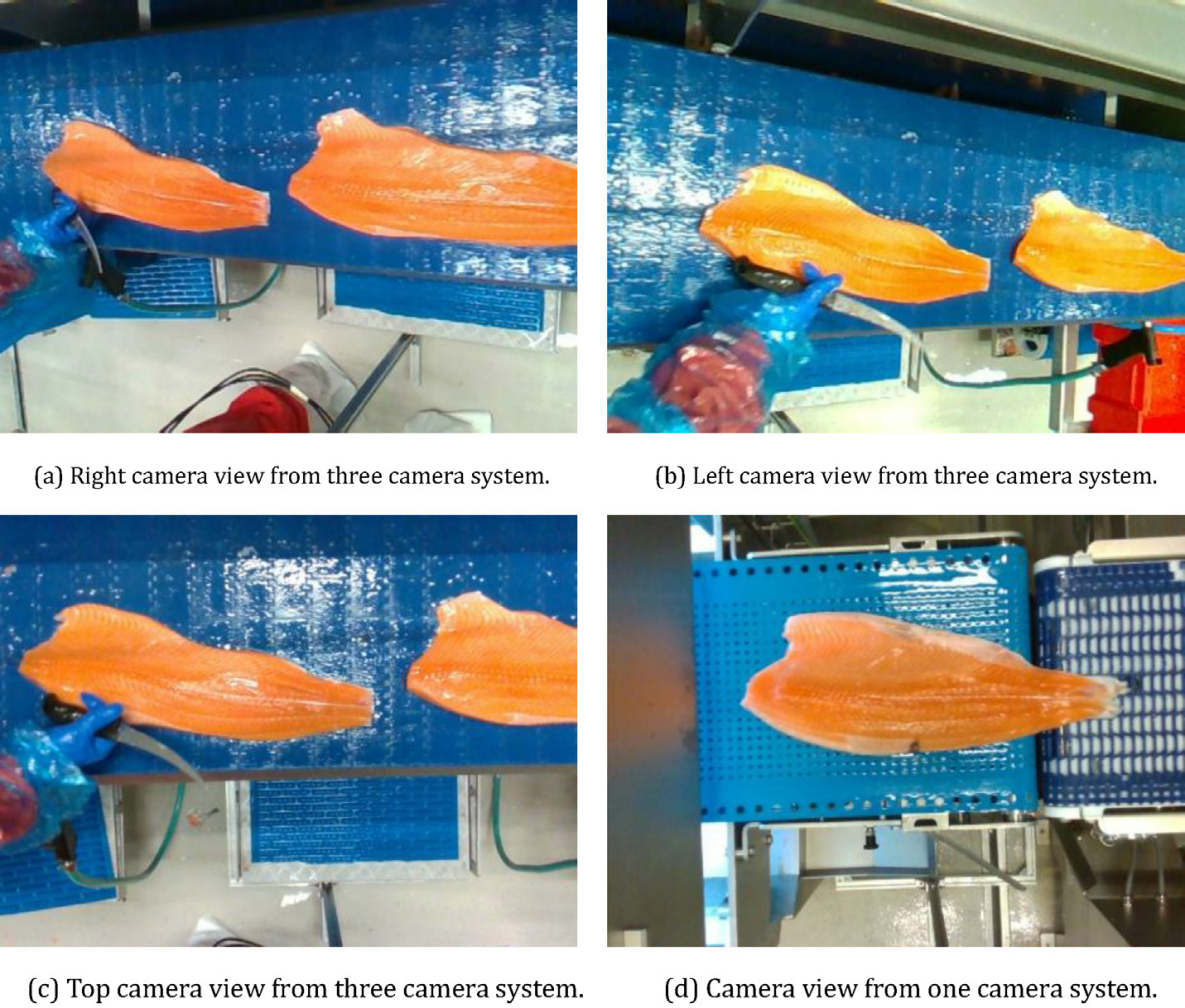
Illustration of various camera views. (a) Right camera view of the trimmed fish fillet, (b) Left camera view of the trimmed fish fillet, (c) Top camera view of the trimmed fish fillet, and (d) Camera view of the untrimmed fish fillet.

The data is collected in the Lerøy Aurora fish processing plant at Skjervøy, in Norway. The data consists of ROS bags and RGB images. The ROS bags also have depth and RGB image information. To record the data, the three-camera setup was placed after the trimming machine, where the fillets were trimmed. A single or one camera system has been mounted between the filleting and trimming machines because the space available was limited to accommodate the three-camera system, so one camera system was employed to record the data of the untrimmed fish fillets. The camera views from each camera are shown in Fig. 1.

### 3.1 *ROS bags descriptions*

A bag is a file type in ROS for storing ROS message data, named after the .bag extension, which plays a significant role in ROS. A variety of approaches are available for storing, processing, analyzing, and visualizing ROS bags. There are 10 ROS bags named from *640_480_60_1.bag* to *640_480_60_10.bag*, recorded with the three camera settings (described in the Experimental Design, Materials and Methods Section) and 5 ROS bags named from *640_480_60_11.bag* to *640_480_60_15.bag* have been recorded from one camera system (with just the top camera as described in the Experimental Design, Materials and Methods Section). The naming structure of these files indicates that the resolution is 640×480 with 60 frames per second while the exposure, white balance and brightness were set to automatic, see Table 1.

**Table 1:** List of available topics from the three camera setting recorded ROS bags.

Topics	Message Type
/camera14_right/color/camera info	Sensor_msgs/CameraInfo
/camera14_right/color/image raw	Sensor_msgs/Image
/camera14_right/depth/camera info	Sensor_msgs/CameraInfo
/camera14_right/depth/image rect raw	sensor_msgs/Image
/camera15_left/color/camera info	Sensor_msgs/CameraInfo
/camera15_left/color/image raw	Sensor_msgs/Image
/camera15_left/depth/camera info	Sensor_msgs/CameraInfo
/camera_top_view/color/camera info	Sensor_msgs/CameraInfo
/camera_top_view/color/image raw	Sensor_msgs/Image
/camera_top_view/depth/camera info	Sensor_msgs/CameraInfo

The ROS bags from these camera settings contain recorded topics, and Table 2 has all the information available on the topics. The right camera captured the color and depth raw image, which can be used for image processing and decision making. Both the top and left cameras have captured raw color images. For analysis, the same procedure can be used to retrieve data information from the single camera configuration.

**Table 2:** Table summarising the dataset.

Number of files	Camera Setup	Data Type	Annotation	Camera Type
10 ROS bags	3 camera setups	ROS bag files	No Annotation	Intel RealSense RGB-D (D415 and D435)
5 ROS bags and 712 RGB images	1 camera setup	ROS bags files and RGB image files	No Annotation	Intel RealSense RGB-D (D435)
509 images	Multispectral Camera (1 camera setup)	RGB.JPG, REG.TIF, RED.TIF, NIR.TIF, GRE.TIF image files	No Annotation	Sequoia Multispectral Camera
32 images (for Data in brief paper data processing)	1 camera setup	RGB image files	Annotated	Intel RealSense RGB-D (D435)
4 JSON files	1 camera setup	.json files	Annotated	Intel RealSense RGB-D (D435)

In addition to the bag files, a separate folder with 712 RGB images of the untrimmed fish fillets is included, which can be utilized for further image processing and decision-making projects. Furthermore, multispectral images captured from the sequoia camera of the fish fillets with melanin and blood spots are available for future research and development.

Additionally, the annotated file that we used to handle the data for this paper is provided. Files with annotations are in the .json format. The annotation of the fish fillet’s belly, melanin, and blood spots is included in four .json files that we provided. We have annotated the data using the VGG annotator tool [10,11]. These four files have the names train_belly.json, train_all.json, val_belly.json, and val_all.json. These JSON files are kept in the train and validation folders. The images used to train the AI model are as well stored in the train and validation folders.

**Fig. 2:**
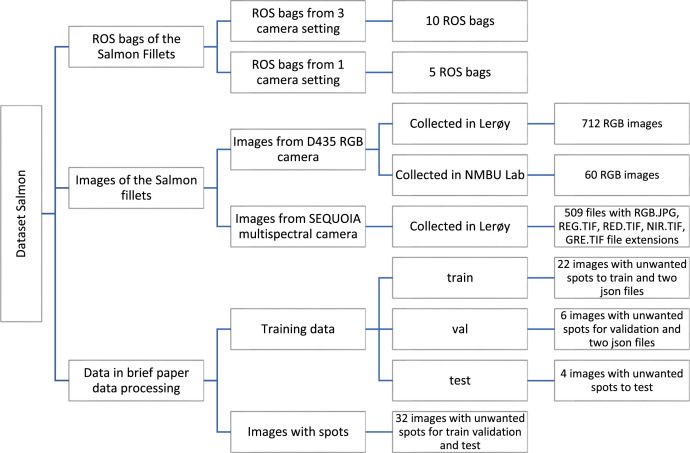
The dataset directory tree.

A full directory tree is presented in Fig. 2 below for a clear understanding of the dataset description.

The following table (Table 2) presents the information about the dataset.

## 4 Experimental Design, Materials and Methods

### 4.1 *Camera system design*

**Fig. 3:**
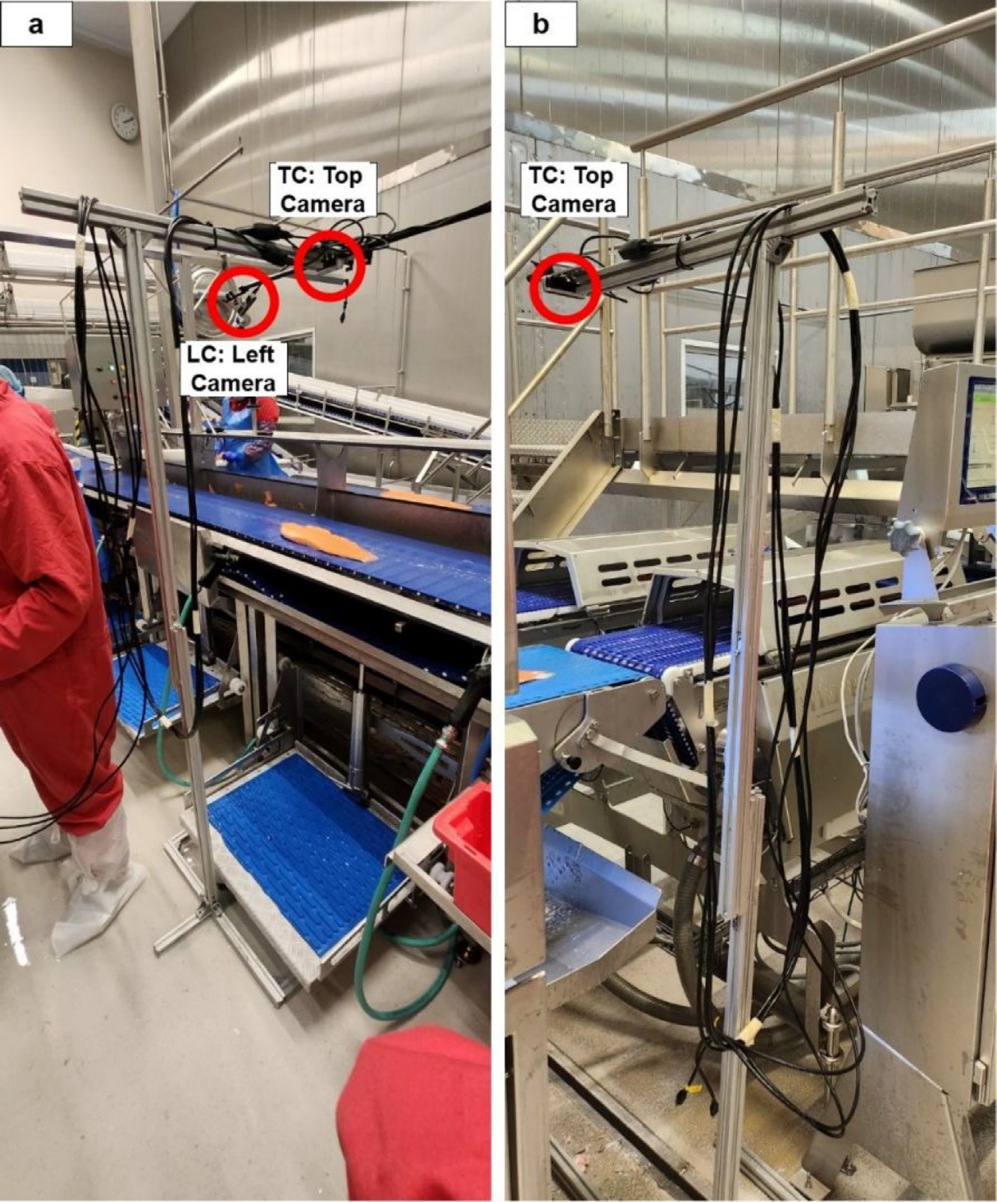
Camera setup stand near the production line (a) 3 camera system (right camera is not in the frame) (b) 1 camera system.

As illustrated in Fig.3 (a, and b), the camera stand comes with an aluminum profile stand. The top camera in the middle was positioned 0.5 m from the left and right cameras and the entire stand measured 1.8 m The cameras on the left and right are Intel Realsense D415 model, while the camera in the middle is an Intel Realsense D435 model. The ROS bags and images are captured using the camera setting shown in the figure. This camera setup was installed near the production line in the industry, and the views these cameras can capture can also be seen in Fig. 1 (a, b, c, and d). This configuration gives the best view of the salmon fillets on the conveyor belt. The single-camera setup was built just by using the top camera (TC), allowing the system with the stand to fit between the filleting machine and trimming machine.

## 5 Materials and Methods

In this section, we will provide a brief data processing overview using the ROS bags and images of this dataset. A MATLAB snippet is provided below that loads a ROS bag file and stores the first 50 RGB images from the bag file.



An important stage in image processing and computer vision is feature mapping. The process of finding and linking significant features in images for a variety of applications, such as object detection, recognition, tracking, and 3D reconstruction, is known as feature mapping in image processing. These features serve as distinctive points that can be used for further analysis and image transformations.

**Fig. 4:**
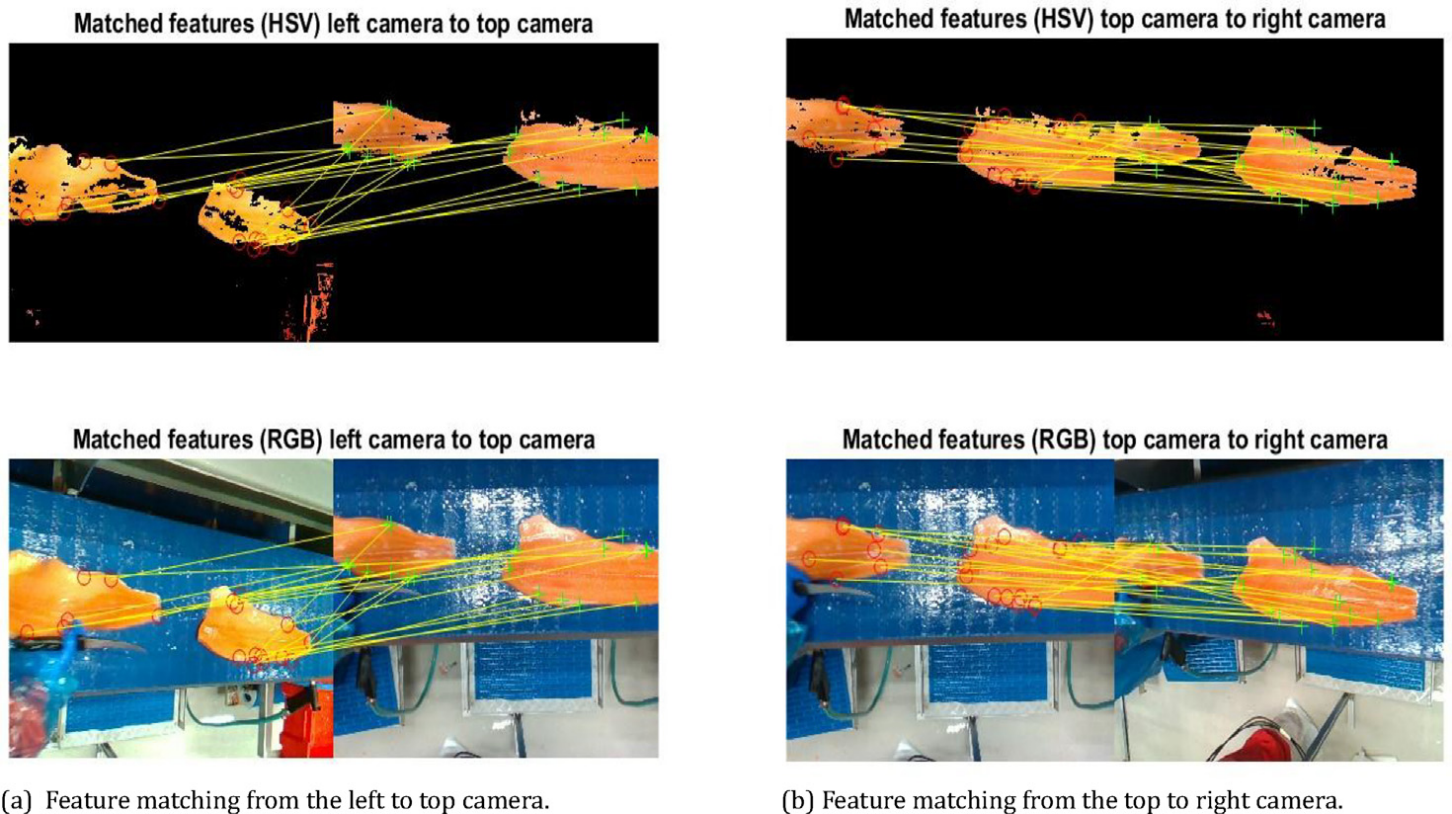
Feature matching from the camera views; (a) left camera to the top camera, (b) top camera to the right camera.

It is extensively employed in fields including robotics, medical imaging, and computer vision. We have used the MATLAB snippet provided in this subsection on the *640_480_60_2.bag* file from the dataset to extract the image and perform feature matching. Fig. 4, shows the matching of features from the left camera to the top camera in the center and the top camera to the right camera.

**Fig. 5:**
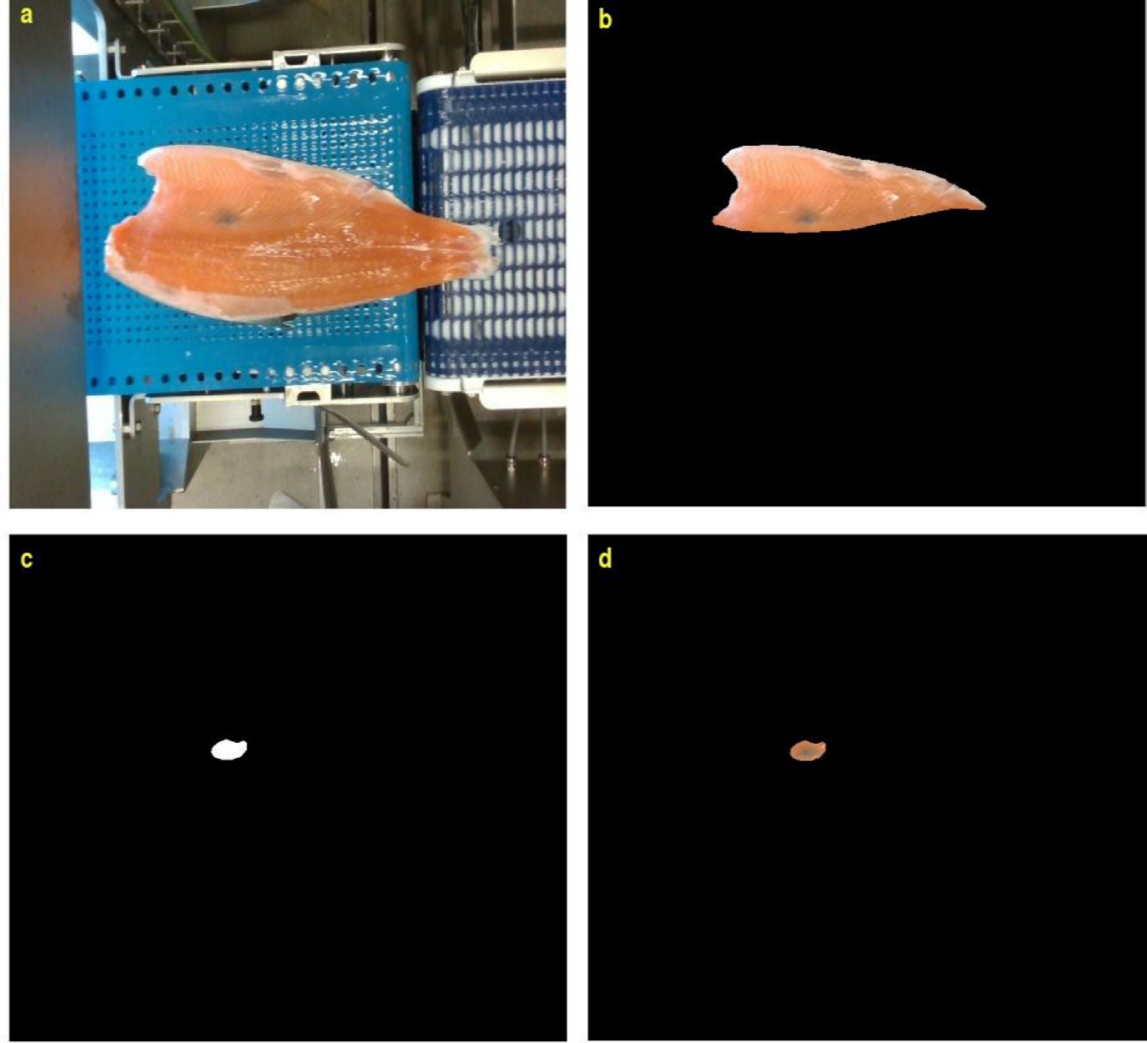
Detection and segmentation of melanin spots. (a) full fillet image (b) belly image. (c, d) the melanin spots.

**Fig. 6:**
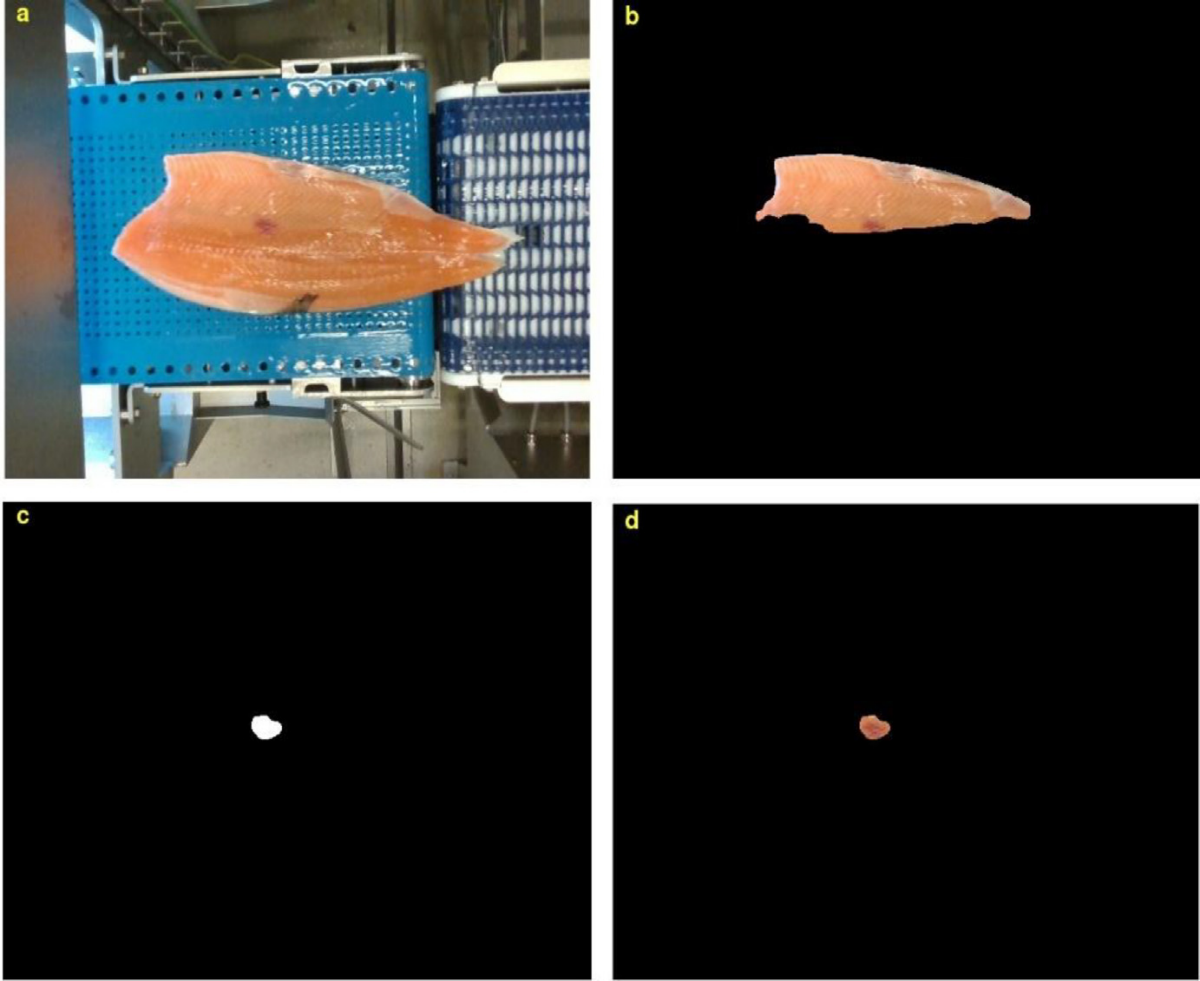
Detection and segmentation of blood spots. (a) full fillet image, (b) belly image, (c, d) the blood spots.

We have also chosen 32 images of the fillets with blood/melanin spots from the 712 raw RGB image dataset to identify the belly area, melanin, and blood spots (Fig. 5 & 6). Recent research indicates that the origins of red and black spots differ, with only black spots containing melanin [12]. Research from the Norwegian Institute of Food, Fisheries, and Aquaculture Research (Nofima) [5] suggests that fillets with high DHA fatty acid concentrations are healthier and have fewer unwanted melanin spots.

We used Detectron2 [13], a deep learning- based object detection and segmentation framework, to train our model to detect and segment the belly and the unwanted spots.

**Fig. 7:**
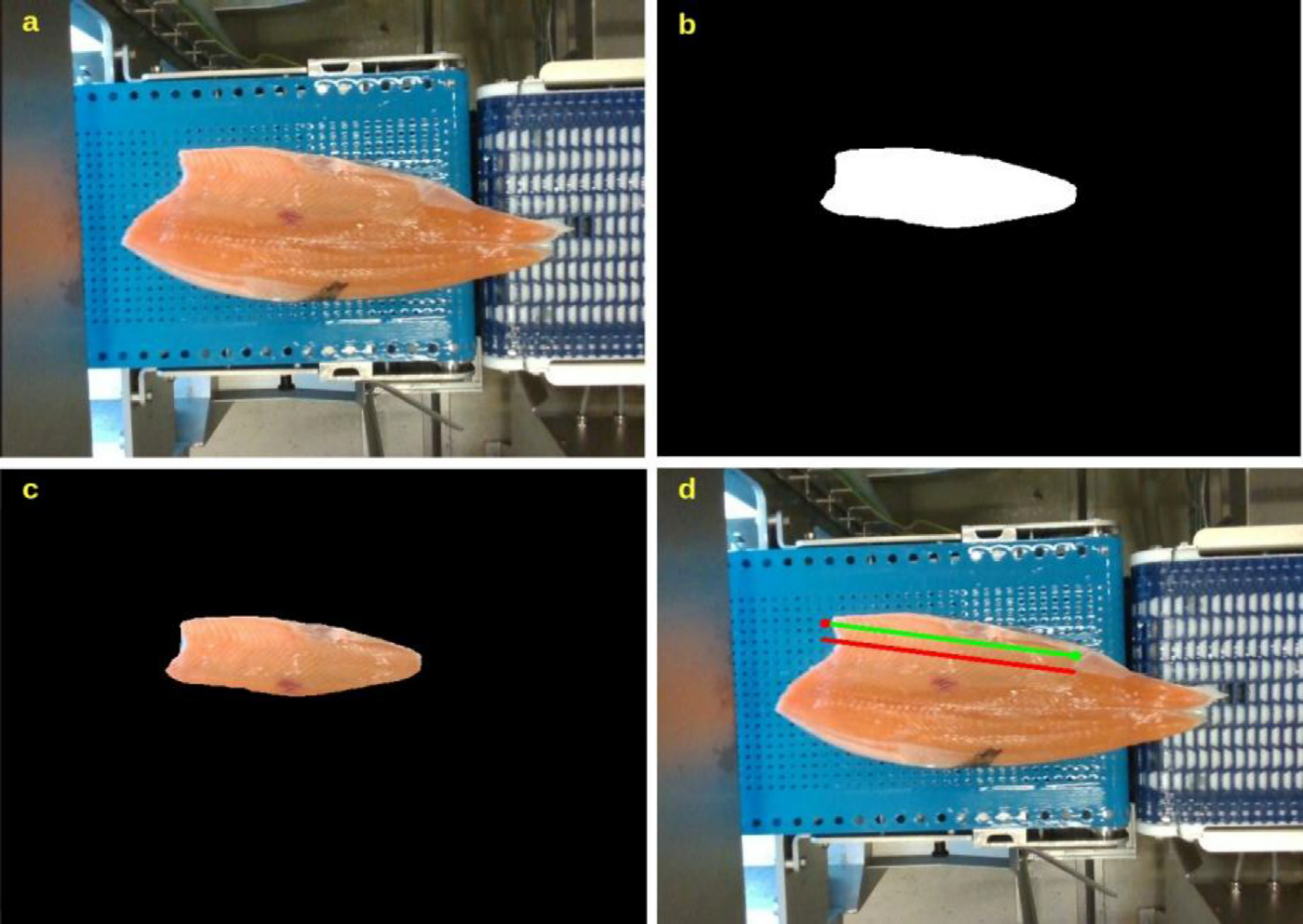
Belly line image processing; (a) fillet on a conveyor belt, (b & c) belly detection and segmentation, (d) scan line on the belly. The red line represents the midline of the belly, while the region below this line, near the white portion of the fillet, has the highest likelihood of providing accurate Omega-3 fatty acid measurements. The green line represents the highest likelihood of providing accurate Omega-3 fatty acids.

The highest concentration of Omega-3 fatty acids is found in the belly of salmon [14]. There have been successful attempts in the literature to automate the process of measuring the fatty acids in the belly. The provided dataset supports the development of computer vision models capable of identifying this region automatically. Once the belly area is detected, robotic systems such as those demonstrated in [1] and [2] can focus scanning or spectroscopic measurements on this region as shown by Fig. 7, improving both efficiency and accuracy in nutrient estimation. Therefore, the dataset is a crucial resource for training and validating models that enable robots to recognize fillet regions of nutritional interest, automate quality assessment, and optimize inspection processes in industrial settings.

## Limitations

The dataset focuses specifically on Salmon fillets collected within a single industrial processing facility, which may limit the generalizability of the findings to other processing environments or species. Additionally, variations due to seasonal, geographic, or handling differences outside the sampled facility are not represented. While the data quality is high, some measurement inconsistencies may exist due to equipment calibration or operator variability. Furthermore, the current version of the dataset includes a relatively low number of annotated images, particularly for unwanted spots, which may restrict the statistical robustness of certain analyses but still serves as a valuable foundation for methodological development and future expansion.

## Ethics Statement

This study did not involve human participants or live animal experimentation. The data were collected from Salmon fillets processing facility during routine industrial operations. As such, no ethical approval or informed consent was required.

## CRediT Author Statement

**Abhaya Pal Singh**: Conceptualization, Data collection and curation, Investigation, Methodology, Writing – original draft; **Michael Angelo Amith Fenelon**: Conceptualization, Data collection and curation, Investigation, Software; **Jens Petter Wold**: Conceptualization, Resources, Supervision, Project administration, Funding acquisition; **Antonio Candea Leite**: Conceptualization, Resources, Supervision, Project administration, Funding acquisition, Writing – review & editing.

## Data Availability

DataverseLaksData: Comprehensive database of salmon fillets images for use in vision-based or food automation applications (Original data).
